# Impact of response bias in three surveys on primary care providers’ experiences with electronic health records

**DOI:** 10.1093/jamia/ocae148

**Published:** 2024-06-18

**Authors:** Nathaniel Hendrix, Natalya Maisel, Jordan Everson, Vaishali Patel, Andrew Bazemore, Lisa S Rotenstein, A Jay Holmgren, Alex H Krist, Julia Adler-Milstein, Robert L Phillips

**Affiliations:** American Board of Family Medicine, Lexington, KY 40511, United States; Center for Professionalism and Value in Health Care, Washington, DC 20036, United States; Division of Clinical Informatics and Digital Transformation, University of California, San Francisco, San Francisco, CA 94117, United States; Department of Health and Human Services, Washington, DC 20201, United States; Department of Health and Human Services, Washington, DC 20201, United States; American Board of Family Medicine, Lexington, KY 40511, United States; Center for Professionalism and Value in Health Care, Washington, DC 20036, United States; Division of Clinical Informatics and Digital Transformation, University of California, San Francisco, San Francisco, CA 94117, United States; Division of Clinical Informatics and Digital Transformation, University of California, San Francisco, San Francisco, CA 94117, United States; Family Medicine & Population Health, Virginia Commonwealth University, Richmond, VA 23219, United States; Division of Clinical Informatics and Digital Transformation, University of California, San Francisco, San Francisco, CA 94117, United States; American Board of Family Medicine, Lexington, KY 40511, United States; Center for Professionalism and Value in Health Care, Washington, DC 20036, United States

**Keywords:** surveys, survey methodology, response bias, physician experience, government data

## Abstract

**Objective:**

To identify impacts of different survey methodologies assessing primary care physicians' (PCPs’) experiences with electronic health records (EHRs), we compared three surveys: the 2022 Continuous Certification Questionnaire (CCQ) from the American Board of Family Medicine, the 2022 University of California San Francisco (UCSF) Physician Health IT Survey, and the 2021 National Electronic Health Records Survey (NEHRS).

**Materials and Methods:**

We evaluated differences between survey pairs using Rao-Scott corrected chi-square tests, which account for weighting.

**Results:**

CCQ received 3991 responses from PCPs (100% response rate), UCSF received 1375 (3.6% response rate), and NEHRS received 858 (18.2% response rate). Substantial, statistically significant differences in demographics were detected across the surveys. CCQ respondents were younger and more likely to work in a health system; NEHRS respondents were more likely to work in private practice; and UCSF respondents disproportionately practiced in larger academic settings. Many EHR experience indicators were similar between CCQ and NEHRS, but CCQ respondents reported higher documentation burden.

**Discussion:**

The UCSF approach is unlikely to supply reliable data. Significant demographic differences between CCQ and NEHRS raise response bias concerns, and while there were similarities in some reported EHR experiences, there were important, significant differences.

**Conclusion:**

Federal EHR policy monitoring and maintenance require reliable data. This test of existing and alternative sources suggest that diversified data sources are necessary to understand physicians’ experiences with EHRs and interoperability. Comprehensive surveys administered by specialty boards have the potential to contribute to these efforts, since they are likely to be free of response bias.

## Introduction

The United States Centers for Disease Control and Prevention began conducting the National Electronic Health Records Survey (NEHRS) with funding from the Office of the National Coordinator for Health IT in 2012 to assess rates of electronic health record (EHR) adoption and patterns of EHR use across office-based physicians in the United States.^1^ It has been conducted almost annually since. Researchers and policymakers have used NEHRS to evaluate and understand how changes in EHR adoption and other healthcare information technologies (HIT) impact clinical practice. More recently, policymakers have used NEHRS to examine office-based physicians’ engagement in electronic exchange of health information and interoperability of EHR systems.^2^ Assessing these HIT functions is important to the Medicare Promoting Interoperability Program and to the strategic objectives of the 21st Century Cures Act.^3^

Despite the important information NEHRS has provided to date about clinicians’ transition from paper to electronic records, there are potential threats to its generalizability. Surveys of physicians and other professionals often suffer from low response rates, raising the possibility of response bias. Response rates in recent years of NEHRS have been as low as 25%, perhaps reflecting well-known challenges in recruiting healthcare professionals to complete surveys.^4^ Similar response rate challenges are common and particularly acute among surveys on informatics topics, and part of a broader trend towards low response rates on surveys of health professionals.^5^ Still, federal policy surveys generally target a response rate of 50% to minimize risk of response bias.^6^^,^^7^ No research to date has focused on the potential impacts of declining response rates on the findings of NEHRS.

Physicians in primary care spend more time documenting care than other physicians and also coordinate care for their patients with other specialists, so it is vital to have high quality data sources about how they use EHRs.^8^ In particular, it is important to find policies that maximize the benefits of EHRs while minimizing their potential to add to physicians’ burdens.^9^^,^^10^ Thus, in this study, we compared primary care physicians’ (PCPs’) responses to three surveys, each intended to gather information on physicians’ use of EHRs but fielded with substantially different strategies: (1) the 2021 NEHRS; (2) the 2022 Continuous Certification Questionnaire (CCQ) from the American Board of Family Medicine (ABFM); and (3) the inaugural version of University of California, San Francisco (UCSF) Physician Health IT Survey, which was also fielded in 2022. The NEHRS was a voluntary survey fielded among a relatively small sample with repeated mailing and active searches to identify the address and contact information for targeted respondents. The CCQ was a required component of Family Medicine physicians’ recertification process through ABFM. The UCSF Physician Health IT Survey was a voluntary survey designed to maximize number of responses under a fixed budget with less focus on response rates.

Our primary aim was to explore policy-relevant differences in respondents and their responses across the three surveys for PCPs’. Specifically, we sought to identify differences in responding physician characteristics and their reported experience with interoperability of health information, as this topic has high policy relevance, was common across all three surveys, and was assessed using comparable questions. The study was designed to improve understanding of the reliability of data used to monitor the effectiveness of policies aiming to improve EHR implementation, use, and interoperability, which are important to patient care. Understanding the strengths and limitations of different methods for ascertaining these policies’ impacts on physicians is critical to inform policymakers, regulators, EHR vendors, and health system leaders working to improve EHR functionality, reduce burden, and improve care.

## Methods

### Population and instruments

We compared responses to the 2021 NEHRS, the 2022 CCQ, and the 2022 UCSF Physician IT Survey (Table 1) covering physicians’ experiences with EHRs.

**Table 1.** Comparison of survey processes.

	ABFM CCQ	UCSF physician health IT survey	NEHRS
Number of respondents	3991	1375 primary care; 3209 total	858 primary care; 1875 total
Response rate	100%	3.6%	18.2%^a^
Survey frame	ABFM Diplomates	Physicians with a listed email in Definitive Healthcare data	American Medical Association and American Osteopathy Association Master files
Breadth of specialties	Family Medicine Physicians	Random sample of all specialties providing direct patient care	Stratified sample of all specialties providing direct patient care
Strategies to garner response rate	Required for continuation of certification	Voluntary survey consisting of repeated contact; 8 emails, 0-2 mailings, 0-1 postcard depending on condition	Voluntary survey consisting of repeated contact (7 emails, 4 mailings and 1 postcard) and tracing—manually identifying contact information.
Survey length and burden	2.5-page survey per respondent (participants randomized to modules)	6-page survey	4-page survey

Abbreviations: ABFM CCQ, American Board of Family Medicine Continuous Certification Questionnaire; NEHRS, National Electronic Health Record Survey; UCSF, University of California, San Francisco.

a This differs from the response rate reported in NEHRS public documentation and reflects the number of completed responses by eligible respondents divided by the total number contacted, excluding physicians who were identified as ineligible.

Physician informants asked to complete the NEHRS were selected through a random recruitment process from the American Medical Association and American Osteopathy Association Master Files, stratified by specialty and region.^4^ They were initially sent both mail and electronic recruitment letters and, subsequently, mail and electronic versions of the survey. The survey included questions intended to assess eligibility for participation, specifically, participants were required to spend most of their working time providing patient care and not be federally employed or more than 85 years of age at the time of the survey. Although NEHRS samples physicians in primary care, specialty care, and surgery, we included only PCPs’ in this analysis and excluded any respondent who said that they do not use an EHR. Each NEHRS respondent was assigned a sample weight based on region and specialty in order to generalize their responses to the population of physicians.

Completion of the CCQ has been required of all family physicians participating in continuous certification processes for more than 30 years.^11^ As such, it has a 100% response rate from a cross-section of nearly 105 000 family physicians. For more than a decade the ABFM has included questions about EHR Meaningful Use policies. For the 2022 CCQ, ABFM collaborated with the United States Office of the National Coordinator for Health Information Technology to incorporate questions about EHR use that closely parallel those in NEHRS. The goal of this collaboration was to enable comparison and to evaluate the potential utility of supplementing NEHRS with outside data. All respondents to the CCQ first answered a set of personal, practice, demographic questions, as well as basic questions about EHR usage, type, and satisfaction. Respondents were then randomized to one of two modules on EHR usage, including one on interoperability. Finally, they were randomized to one of five modules that covered topics such as burnout and meaningful use of EHRs. Thus, a few EHR questions are answered by all ABFM Diplomates, each of two modules are answered by 50% of all respondents, and 20% of respondents answer each of five modules. Only respondents who indicated that they use an EHR system and that they provided direct patient care were included in the analysis. To ensure comparability with NEHRS, we also excluded federally employed physicians.

The UCSF Physician Health IT Survey was initiated in 2022 to collect in-depth information on how information technology is integrated into clinical settings. Researchers used simple random sampling to select 90 000 potential participants with listed email addresses from Definitive Healthcare, a proprietary dataset designed for healthcare analytics. All sampled physicians received 8 emails. 60 000 sampled physicians also received a postcard reminder to complete the survey. 30 000 sampled physicians also received 2 recruitment letters by mail. The UCSF survey recruited physicians of all specialties, but only physicians who used EHRs and who worked in primary care for non-federal employers were included in this analysis.

#### Statistical analysis

To account for sample weighting in NEHRS and the UCSF survey, we used a Rao-Scott corrected chi-square test, which adjusts the chi-square estimate for weights to account for changes in the composition of the population.^12^ Since our interests were in identifying specific differences in the surveys, we conducted significance tests in a pairwise fashion across the three surveys.

While the ABFM CCQ and the UCSF survey both based questions on NEHRS, some questions were phrased differently or included different answer options. We did not conduct significance testing on the differences in the responses to questions that we found to be incomparable across surveys, but we did retain them in the tables for reference.

As a supplemental analysis, we also conducted stratified comparisons of especially important indicators of interoperability experience across age groups (less than 50 or 50-plus years), EHR platform (Epic or other), and practice type (private practice or other).

All analyses were conducted in R version 4.1.2, with the Rao-Scott corrected chi-square test implemented in the “survey” package version 4.1.1.^13^^,^^14^

## Results

### Response rate comparison

The CCQ had the highest number of respondents and response rate across the three surveys. A total of 3991 respondents to the 2022 CCQ provided direct patient care and were otherwise eligible for inclusion in the analysis (100% response rate). Of the 10 302 physicians sampled for NEHRS in 2021, 1875 were eligible and completed the survey (18.2% response rate); although we could not determine how many family physicians were in the NEHRS sample, 858 (48.7% of respondents) listed primary care as their specialty.^4^ Among the 90 000 physicians sampled for the UCSF Physician Health IT Survey, 3209 responded (3.6% response rate), among whom 1375 (42.8% of respondents) practiced in primary care.

### Respondent demographics

Respondents to the three surveys represented different physician demographics (Table 2). Respondents to the CCQ were the youngest: the share of respondents aged 35 to 44 (42.1%) was over twice that seen in the NEHRS (17.0%) and the UCSF survey (15.2%). Male and female genders were approximately equally represented in all surveys.

**Table 2.** Survey respondent and practice characteristics.

	ABFM CCQ		UCSF		NEHRS (*n* = 858)	
	*N*	%	Unweighted *N*	Weighted %	Unweighted *N*	Weighted %
**Age**						
Under 35	66	1.7	18	1.3	37	3.6
35-44	1682	42.1	200	15.2	151	17.0
45-54	1224	30.7	333	27.0	263	32.9
55-64	721	18.1	402	33.0	242	26.2
65+	298	7.5	285	23.5	165	20.2
**Gender**						
Female	1960	49.1	672	51.7	384	47.2
Male	2031	50.9	637	48.3	474	52.8
**Which of the following describes your principal practice site**						
Private solo or group practice, or freestanding clinic/urgent care center	1169	29.3	460	39.8	606	73.1
Integrated Delivery System, Health maintenance organization, health system or other prepaid practice (eg, Kaiser Permanente)	1844	46.2	117	7.4	95	11.5
Government clinic that is not federally funded (eg, state, county, city, maternal and child health, etc.)	62	1.6	12	0.9	10	1.4
Academic health center/faculty practice	337	8.4	429	29.2	63	6.4
Federally Qualified Health Center or Look-Alike, or Rural Health Clinic	435	10.9	166	13.4	84	7.6
Other^a^	144	3.6	125	9.3	—^b^	
**Which of the following describes your principal practice size?**						
1-5 Providers	1694	42.4				
6-20 Providers	1229	30.8				
>20 Providers	1068	26.8				
**How many physicians, including you, work at this practice (including physicians at the reporting location, and physicians at any other locations of the practice)?** ^b^						
1 physician			104	9.9	163	21.3
2-3 physicians			165	14.4	187	20.7
4-10 physicians			388	30.5	272	29.3
11-50 physicians			319	23.6	147	13.9
>50 physicians			330	21.5	89	14.8
**Practice location**						
Large central metropolitan	1163	31.6	562	41.7	151	34.7
Large fringe metropolitan	858	23.3	217	18.2	156	25.2
Medium metropolitan	853	23.2	229	18.5	227	21.3
Small metropolitan	345	9.4	130	10.7	134	8.7
Micropolitan (non-metropolitan)	279	7.6	82	6.9	117	6.3
Non-core (non-metropolitan)	185	5.0	47	4.2	73	3.7

a The ABFM CCQ and UCSF survey included questions on whether the practice site was Federally owned or part of the Indian Health Service, and these respondents were excluded from all analyses. The NEHRS excludes Federally employed physicians and, therefore, does not capture these practice types. NEHRS excludes physicians practicing in hospital outpatient departments from the survey and these account for 114 of 125 “Other” responses in the UCSF data. They are included in all estimates of UCSF data.

b Fewer than 5 physicians reported Other practice locations on the NEHRS, below the minimum cell size for reporting.

The share of responses by setting varied across the three surveys. A disproportionate share of NEHRS respondents worked in private practice (73.1%) compared to CCQ (39.8%) or UCSF (29.3%). While a plurality of CCQ respondents were from health systems (46.2%), this setting was not well represented in either the USCF (7.4%) or NEHRS (11.5%) surveys. Far more respondents to the UCSF survey—29.2%—practiced in academic health centers or faculty practices.

There was also some variability by practice size. Physicians from practices with more than 10 physicians were overrepresented in the UCSF survey (45.1%) vs NEHRS (28.7%). The CCQ asked about the number of providers working at the respondent’s main practice site instead of physicians at all locations of the practice, which is how NEHRS and UCSF surveys asked the question. Despite this more inclusive clinician language, a larger proportion of CCQ physicians (42.4%) indicated that they work in practices with 1-5 providers. Geographic representation was similar in the CCQ and NEHRS, but respondents to the UCSF survey were more likely to practice in central metropolitan locations compared to the other surveys’ respondents.

### EHR vendor

There were significant differences in the EHR platform used by respondents and their satisfaction with these EHRs across the surveys (Table 3). While Epic was the most common EHR platform in all three surveys, Cerner was the second most commonly used platform in the UCSF survey and eClinical Works in the others. The CCQ had the largest share of respondents indicating that they do not know which EHR they use (1.8%). Respondents to NEHRS reported being very satisfied with their EHR at significantly higher rates (29.1%) than in the CCQ (26.7%) or UCSF survey (19.4%).

**Table 3.** EHR platform and user experience.

	ABFM CCQ		UCSF		NEHRS	
	*N*	%	Unweighted *N*	Weighted %	Unweighted *N*	Weighted %
**EHR vendor** ^a^						
Allscripts	211	5.4	50	5.3	48	5.0
athenahealth	372	9.6	75	8.1	94	9.7
Cerner	288	7.4	122	12.6	66	5.4
eClinical Works	431	11.1	87	9.1	110	13.9
e-MDs	32	0.8	8	0.8	16	2.2
Epic	1576	40.6	743	41.4	233	29.5
NextGen	172	4.4	47	4.8	46	7.2
Practice Fusion	84	2.2	9	0.9	21	3.1
Greenway	66	1.7	22	2.2	36	3.5
Other	582	15.0	139	14.2	183	20.2
Don’t know	68	1.8	6	0.6	2	0.2
**EHR satisfaction** ^b^						
Very satisfied	1028	26.7	276	19.4	229	29.1
Somewhat satisfied	1496	38.9	480	35.3	336	39.1
Neither satisfied nor unsatisfied	314	8.2	114	9.1	77	7.4
Somewhat dissatisfied	630	16.4	281	22.6	130	15.2
Very dissatisfied	377	9.8	158	13.5	80	9.3
**Ease of documenting care (ABFM CCQ and UCSF survey wording in parentheses)** ^a^						
Very easy (Excellent)	420	22.2	202	14.8	134	15.2
Somewhat easy (Good)	882	46.5	546	40.4	402	47.3
Somewhat difficult (Fair)	475	25.1	429	33.6	265	33.7
Very difficult (Poor)	119	6.3	131	11.1	52	3.8

a *P*-values for all comparisons < .001.

b *P*-values for ABFM vs UCSF and NEHRS vs UCSF < .001, *P*-value for ABFM vs NEHRS = .875.

### EHR use

**Figure 1.**
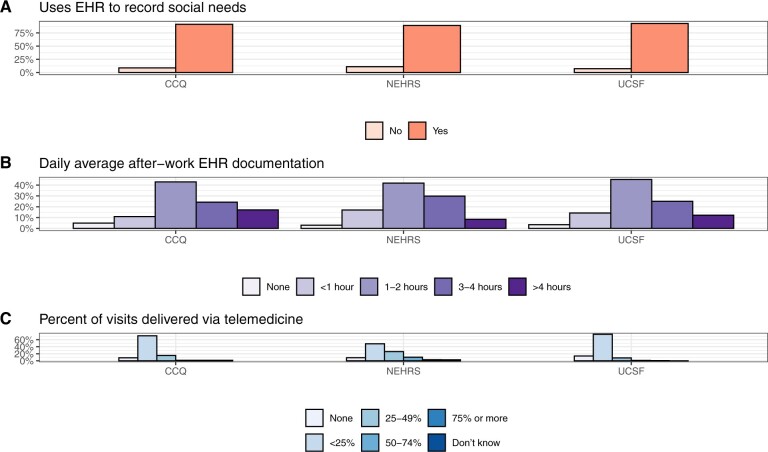
Comparison of documentation and practice patterns across the three surveys. For C, note that the CCQ and UCSF survey ask about telemedicine in the prior 3 months, while NEHRS asks about telemedicine since March 2020.

Similarly, documentation and ease of documentation differed (Figure 1, Supplementary Material I). CCQ respondents reported spending more than 4 hours in after-work documentation at significantly higher rates (17.1%) than respondents in the other surveys (12.2% for UCSF and 8.4% for NEHRS). Interestingly, more CCQ respondents said that their experience of documenting care in the EHR was excellent/very easy (22.2%) vs the others (14.8% for UCSF, 15.2% for NEHRS).

### Interoperability

Several questions related to interoperability were similar across all three surveys; however, response options were identical for only 2 questions assessing 2 items (Table 4). 45.8% of respondents to the CCQ and 45.4% of respondents to NEHRS indicated that their EHRs integrate patient health information from outside organizations into their EHR. 22.8% of respondents in CCQ and 21.9% of respondents to NEHRS indicated that they often had access to clinical information from outside organizations in their EHR. On both items, respondents to the UCSF survey were substantially less positive, with 37.6% of UCSF respondents indicating that their EHR integrated information and 14.6% indicating that they often had access to clinical information.

**Table 4.** Interoperability experience.

	ABFM CCQ		UCSF		NEHRS	
	*N*	%	Unweighted *N*	Weighted %	Unweighted *N*	Weighted %
**Do you electronically receive patient health information from other providers outside your organization via EHR or web portal?** ^a^						
Often	824	42.3	297	21.2	518	64.6
Sometimes	712	36.6	427	31.0		
Rarely	200	10.3	244	19.4		
Never	151	7.8	227	20.0	275	29.8
Don’t know	61	3.1	110	8.5	65	5.6
**Does your EHR integrate patient health information received electronically without special effort or manual entry?** ^b^						
Yes	327	45.8	430	37.6	353	45.4
No	207	29.0	499	37.0	344	36.7
Don’t know	180	25.2	364	25.4	161	17.9
**When seeing a new patient, do you electronically query for the patient’s health information outside your organization?** ^a^						
Often	299	40.0	568	38.1	487	54.4
Sometimes	179	24.0	277	20.2		
Rarely	111	14.9	185	15.9		
Never	94	12.6	243	23.7	337	40.1
Don’t know	64	8.6	29	2.1	34	5.5
**When seeing patients treated by clinicians outside your organization, how often do you have clinical information from those outside encounters electronically available in your EHR?** ^a^						
Often	170	22.8	219	14.6	185	21.9
Sometimes	290	38.8	534	35.8	371	41.5
Rarely	130	17.4	273	23.2	160	19.4
Never	72	9.6	237	23.6	111	13.1
Don’t know	58	7.8	25	1.8	18	1.5
Do not see patients from outside organization	27	3.6	12	0.9	13	2.6

a *P*-values for all comparisons < .001.

b *P*-value for ABFM vs NEHRS and ABFM vs UCSF < .001, *P*-value for NEHRS vs UCSF = 0.0.

Response options differed between NEHRs and the CCQ and UCSF survey for 2 additional interoperability options, making direct comparison across surveys more challenging. For one of these items, respondents to the CCQ had a better experience of interoperability than respondents to the UCSF survey: 42.3% of CCQ respondents said that they often receive external information via EHR or web portal, compared to 21.2% of UCSF respondents. 64.6% of NEHRS respondents said that they received information but were not asked how often. For the second item, responses to the CCQ and UCSF were more similar: 40.0% of respondents to the CCQ indicated that they often queried for information from outside organizations for new patients compared to 38.1% of respondents to the UCSF survey. 54.4% of respondents to the NEHRS indicated that they queried for information, but again were not asked how often.

Some additional questions about the ease of using interoperable data were only asked in the CCQ and UCSF survey (Supplemental Material II). As before, UCSF respondents indicate more negative experiences with EHRs. For example, 24.1% of CCQ respondents say it is very easy to include external information in care decisions compared to 18.6% of UCSF respondents.

### Stratified comparisons across surveys

**Figure 2.**
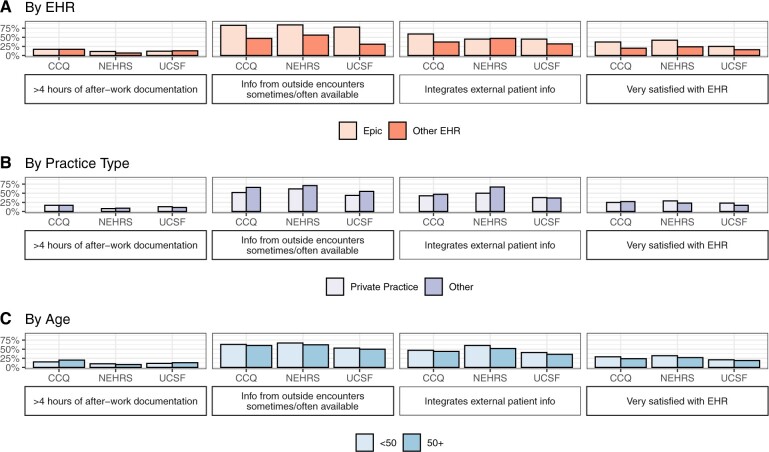
Stratified comparisons of key interoperability and EHR satisfaction measures.

We designed our stratified analysis to identify whether differences in survey composition—such as a greater number of CCQ respondents in the youngest age group—explained observed differences in opinions or if these differences persisted within sub-groups (Figure 2, Supplemental Material III). Results indicated that survey differences persisted across sub-groups: for instance, both older and younger respondents to the CCQ were more likely to say that information was integrated into their EHR than were the same age group respondents to the UCSF survey.

Differences between sub-groups were directionally consistent across most comparisons in the three surveys. For instance, all 3 found that Epic users were more likely to be very satisfied with their EHR, perhaps due in part to the higher interoperability ratings for Epic found across surveys. All three surveys also concurred that physicians in private practice were less likely to have external patient health information available, and that physicians aged 50 years or older were less likely to integrate external information into care decisions.

## Discussion

These findings reflect a rare opportunity to compare self-reported experiences with health IT across multiple surveys with different sampling strategies. While the ABFM CCQ is a cross-sectional census with 100% response rate from a single, large specialty, physicians are less likely to respond to discretionary, voluntary surveys such as NEHRS and UCSF, potentially making them less representative of the physician population at large. Comparing these three surveys expands our understanding of their validity, reliability, and generalizability and has implications for the interpretation of voluntary surveys on informatics more generally. This comparison indicates that, relative to the CCQ, respondents to NEHRS and UCSF are not representative of PCPs’, and thus their responses on IT related questions may not reflect the views of this population. Comparison of responses indicates that responses to the UCSF survey, in particular, were less positive than responses to either the NEHRS or CCQ.

Differences in response rate, sampling strategy and respondent characteristics across the surveys notwithstanding, the findings across surveys showed some broad similarities. Across surveys, only about 20% of physicians reported that information was often available from their patients’ healthcare encounters outside the primary care clinic. The 3 surveys also indicated that users of Epic were more likely to have this information available, especially from other systems using Epic, and that physicians working in private practice were less likely to. Other research has found that practice site and physician characteristics alone do not account for the high satisfaction rates with Epic compared to other EHRs; rather, it seems that usability and interoperability may be the dominant reasons why physicians report higher satisfaction with Epic.^15^

Despite these common findings, almost all questions had statistically significant differences across surveys. Some of these differences in findings between the CCQ and the other surveys may lead to different policy implications and level of urgency to address these challenges. For instance, relative to NEHRS, nearly twice as many CCQ respondents indicated spending more than 4 hours a day documenting outside of work, and relative to CCQ, about half as many UCSF respondents indicated that they often received information from outside organizations. In these cases and several other questions where responses to NEHRS and CCQ broadly concurred, responses to the UCSF survey represented more negative experiences with EHRs.

These differences may have been driven by a combination of observed and unobserved differences in respondents brought about by differing response rates and survey strategies. The plurality of CCQ respondents were aged 35-44, younger than in the other surveys. They were also over four times as likely to work at a health system compared to UCSF and NEHRS respondents, which were far more likely to work in academic and independent practices, respectively. The composition of CCQ practices more closely resembles data reported by others.^16^

While differences in demographics alone do not necessarily imply bias in views on substantive questions,^17^ we found differences in views on health IT across surveys that persisted among age, EHR vendor, and practice type subgroups. These persistent differences were likely attributable to unobserved differences in respondents and non-respondents across surveys. These findings have important implications for surveys of health professionals about informatics beyond these 3 surveys because response rates similar to the UCSF survey are not uncommon in the informatics literature.^18–21^

Our results provide some guidance in interpreting response bias in these surveys, including those planned by the AMIA 25 × 5 Task Force,^22^ by indicating respondents to such surveys may have motivations that are not well captured by observable characteristics. Given widespread dissatisfaction with health IT by health professionals, it is likely that respondents have specific concerns and may provide more negative responses than the population at large. In our data, this may have driven results among respondents to the UCSF survey in particular.

Our findings suggest that certifying boards, such as ABFM, could be important partners in providing data that inform EHR policies and the benefits and burdens these have for the clinicians they certify. The data are likely to have higher representativeness and reliability, especially as a mandatory portion of recertification activities. Broader participation by boards would expand understanding of the EHR experience of physicians within particular medical specialties. Certifying boards are also able to invite the physicians they certify to provide qualitative information about emerging issues with EHRs and other medical technologies that can further inform policy and build better surveys. Certifying board’s questionnaires are also faster to collect than voluntary surveys, offering more rapid evaluation of policy impacts. We respect that recertification questionnaires as a forced function are also burden, but the potential for improving EHR functionality, reducing burden, and improving care are real and meaningful.

### Limitations

There were several limitations to this work. Despite coordination between the three institutions, there were some differences in the questions or their response options. Some questions, such as the question about practice size, had been carried forward from previous versions of the CCQ. This limited our ability to reach more detailed conclusions about some potential comparisons. Perhaps more substantially, the CCQ only included physicians within one subspecialty of primary care, albeit the third largest of all medical specialties. There are some differences between family physicians and other types of PCPs’, including that family physicians are more likely to practice in rural and underserved areas.^23^ In many other ways, though, family physicians are quite similar to other physicians working in primary care.^24^^,^^25^ As such, we believe that their experiences are generalizable across primary care although this could only be validated with the support of surveys of other specialties.

## Conclusion

Our study compared three surveys of EHR experience among physicians in primary care to understand the potential impact of response bias and survey methodology on policy-relevant outcomes. We found that while similar conclusions could be drawn at a high level related to the state of interoperability, there were potentially important differences in the demographics of respondents: specifically, voluntary surveys such as NEHRS are less likely to capture individuals experiencing especially high documentation burdens and may thus overstate EHR satisfaction. These differences are meaningful for policymakers whose objectives are to advance the development and use of HIT and improving data sharing in service to patient care. Diversified data sources are necessary to arrive at the reliable data they need to create and monitor these policies. Comprehensively sampled surveys using instruments such as those administered by specialty boards are crucial complements to current, voluntary surveys to overcome sampling bias and achieve more granular understanding of clinician EHR experience. Future work is needed to assess variation in self-reported HIT experiences of physicians in other medical subspecialties and to understand how potentially countervailing forces in CCQ and NEHRS could have produced similar responses despite meaningfully different respondent panels. Physician certifying boards may find common interest in reducing EHR burden and improving EHR functionality for their Diplomates as a part of their commitment to supporting the profession and patients.

## Supplementary Material

ocae148_Supplementary_Data

## Data Availability

The NEHRS data used in this research are publicly available through the website of the United States Centers for Disease Control and Prevention (Office of the National Coordinator for Health Information Technology. NEHRS Dataset Documentation [Internet]. [cited August 8, 2022]. https://ftp.cdc.gov/pub/Health_Statistics/NCHS/Dataset_Documentation/NEHRS/). ABFM CCQ data may be accessed for IRB-approved projects subject to the approval of the ABFM Research Governance Board; please contact the corresponding author for details. The UCSF survey data underlying this article cannot be shared due to privacy restrictions.
