# Supplementary material for: Impact of response bias in three surveys on primary care providers’ experiences with electronic health records
Source: J Am Med Inform Assoc. 2024 Jun 18;31(8):1754–62. doi: 10.1093/jamia/ocae148 (PMC11258403; doi:10.1093/jamia/ocae148)
Supplement: ocae148_Supplementary_Data [file ocae148_supplementary_data.docx]

**Supplemental material for “Comparative Analysis of Three Surveys on Primary Care Providers’ Experiences with Interoperability in Electronic Health Records”**

1. *Documentation and visit practices*

|  | **ABFM CCQ** | | **UCSF** | | **NEHRS** | |
| --- | --- | --- | --- | --- | --- | --- |
|  | **N** | **%** | **Unweighted N** | **Weighted %** | **Unweighted N** | **Weighted %** |
| *Uses EHR to record social determinants of health** | | | | | | |
| Yes | 1,620 | 91.3 | 1214 | 92.8 | 687 | 89.1 |
| No | 155 | 8.7 | 95 | 7.2 | 81 | 10.9 |
| *Daily average of after-work EHR documentation*** | | | | | | |
| None | 95 | 4.9 | 42 | 3.4 | 36 | 3.0 |
| Less than 1 hour | 211 | 10.9 | 184 | 14.2 | 129 | 17.0 |
| 1 to 2 hours | 830 | 42.9 | 598 | 45.2 | 371 | 41.8 |
| 3 hours to 4 hours | 468 | 24.2 | 326 | 25.0 | 247 | 29.8 |
| More than 4 hours | 330 | 17.1 | 157 | 12.2 | 75 | 8.4 |
| *Percent of patient visits delivered via telemedicine (ABFM CCQ and UCSF: in last 3 months, NEHRS: since March 2020)^***^* | | | | | | |
| None | 168 | 8.7 | 166 | 13.9 | 81 | 8.8 |
| Less than 25% | 1375 | 71.1 | 993 | 75.4 | 451 | 48.3 |
| 25% to 49% | 293 | 15.1 | 119 | 8.5 | 212 | 26.2 |
| 50% to 74% | 33 | 1.7 | 17 | 1.2 | 69 | 10.3 |
| 75% or more | 33 | 1.7 | 11 | 0.7 | 29 | 3.2 |
| Don’t know | 33 | 1.7 | 2 | 0.2 | 15 | 3.1 |

**p-value for ABFM versus NEHRS = 0.183, ABFM versus UCSF = 0.138, and UCSF versus NEHRS = 0.023 **p-value for ABFM versus NEHRS and ABFM versus UCSF < 0.001, p-value for NEHRS versus UCSF = 0.094. *** p-values for all comparisons < 0.001*

1. *Integration into care of patient information from external organizations*

|  | **ABFM** | | **UCSF** | |
| --- | --- | --- | --- | --- |
|  | **N** | **%** | **Unweighted N** | **Weighted %** |
| *When you access clinical information from outside your organization (e.g. referrals, consult notes, discharge summaries, patient records) through any means (e.g. fax, phone, EHR, etc), how easy is it to use the information to effectively care for your patients?** | | | | |
| Not at all | 158 | 8.2 | 122 | 18.5 |
| Somewhat | 1270 | 65.9 | 838 | 62.8 |
| Very | 465 | 24.1 | 241 | 18.6 |
| Don't Know | 33 | 1.7 | 2 | 0.1 |
| *When you access clinical information about your patients from outside your organization (e.g. referrals, consult notes, discharge summaries, patient records), how often is it from within your EHR in any integrated format (as opposed to a PDF)?** | | | | |
| Never | 299 | 15.3 | 249 | 23.9 |
| Rarely | 256 | 13.1 | 217 | 17.8 |
| Sometimes | 630 | 32.3 | 398 | 28.1 |
| Often | 618 | 31.7 | 360 | 24.4 |
| Don't Know | 145 | 7.4 | 75 | 5.7 |
| *How easy is it to use clinical information from clinicians outside your organization that use the same EHR Vendor?** | | | | |
| Not at all | 250 | 13.8 | 179 | 19.1 |
| Somewhat | 646 | 35.6 | 459 | 36.3 |
| Very | 765 | 42.2 | 500 | 35.6 |
| Don't Know | 152 | 8.4 | 91 | 9.1 |
| *How easy is it to use clinical information from clinicians outside your organization that use a different EHR Vendor** | | | | |
| Not at all | 615 | 32.6 | 726 | 54.3 |
| Somewhat | 947 | 50.3 | 455 | 36.8 |
| Very | 150 | 8.0 | 41 | 4.0 |
| Don't Know | 172 | 9.1 | 66 | 4.9 |
| *When looking for or using clinical information from outside your organization, to what extent do the following occur: Difficulty finding important information due to a large amount of low-value information** | | | | |
| Not at all | 99 | 5.4 | 25 | 2.1 |
| To Some Extent | 883 | 48.1 | 330 | 24.4 |
| To a Great Extent | 855 | 46.5 | 932 | 73.5 |

**p-value < 0.001*

1. *Stratified comparisons*

| **EHR Vendor** | Epic | | | Other Vendor | | |
| --- | --- | --- | --- | --- | --- | --- |
|  | ABFM | NEHRS | UCSF | ABFM | NEHRS | UCSF |
|  | % (95% CI) | % (95% CI) | % (95% CI) | % (95% CI) | % (95% CI) | % (95% CI) |
| Very satisfied with EHR | 37 (34 – 39) | 42 (31-54) | 25 (22-29) | 20 (18 – 22) | 24 (19-30) | 16 (13-19) |
| More than 4 hours daily after-work documentation | 17 (15 – 19) | 11 (6-21) | 12 (9-14) | 17 (15 – 19) | 7 (5-11) | 13 (10-16) |
| Integrates patient information | 59 (53 – 65) | 78 (63-88) | 45 (41-49) | 37 (32 – 41) | 47 (40-54) | 32 (28-36) |
| Information from outside encounters sometimes/often available | 83 (79 – 87) | 84 (71-92) | 78 (75-81) | 47 (42 – 52) | 56 (49-62) | 31 (28-36) |
| **Practice Site** | Private/Independent Practice | | | Other | | |
| Very satisfied with EHR | 25 (23 – 28) | 29 (23-36) | 23 (19-27) | 27 (26 – 29) | 23 (20-40) | 17 (14-20) |
| More than 4 hours daily after-work documentation | 17 (14 – 20) | 8 (6-12) | 13 (10-17) | 17 (15 – 19) | 9 (4-18) | 11 (9-14) |
| Integrates patient information | 43 (36 – 49) | 50 (43-58) | 38 (33-43) | 47 (43 – 52) | 67 (56-76) | 37 (33-40) |
| Information from outside encounters sometimes/often available | 52 (46 – 59) | 62 (55-68) | 44 (39-49) | 66 (62 – 70) | 71 (60-80) | 55 (51-59) |
| **Age** | <50 | | | 50+ | | |
| Very satisfied with EHR | 29 (27 – 30) | 32 (24-43) | 21 (17-26) | 24 (22 – 26) | 27 (21-34) | 19 (16-22) |
| More than 4 hours daily after-work documentation | 15 (13 – 17) | 10 (5-18) | 11 (8-15) | 20 (17 – 23) | 8 (5-11) | 13 (11-16) |
| Integrates patient information | 47 (42 – 52) | 60 (49-70) | 41 (35-47) | 44 (39 – 50) | 52 (45-60) | 36 (33-40) |
| Information from outside encounters sometimes/often available | 63 (59 – 68) | 67 (58-76) | 53 (47-59) | 60 (54 – 65) | 62 (55-69) | 50 (46-53) |
